# A modulated empirical Bayes model for identifying topological and temporal estrogen receptor α regulatory networks in breast cancer

**DOI:** 10.1186/1752-0509-5-67

**Published:** 2011-05-09

**Authors:** Changyu Shen, Yiwen Huang, Yunlong Liu, Guohua Wang, Yuming Zhao, Zhiping Wang, Mingxiang Teng, Yadong Wang, David A Flockhart, Todd C Skaar, Pearlly Yan, Kenneth P Nephew, Tim HM Huang, Lang Li

**Affiliations:** 1 Center for Computational Biology, Indiana University School of Medicine, Indianapolis, IN 46202, USA; 2 Division of Biostatistics, Indiana University School of Medicine, Indianapolis, IN 46202, USA; 3 Department of Medical and Molecular Genetics, Indiana University School of Medicine, Indianapolis, IN 46202, USA; 4 Division of Clinical Pharmacology, Indiana University School of Medicine, Indianapolis, IN 46202, USA; 5 Indiana University Melvin and Bren Simon Cancer Center, Indiana University School of Medicine, Indianapolis, IN 46202, USA; 6 Center for Medical Genomics, Indiana University School of Medicine, Indianapolis, IN 46202, USA; 7 Departments of Cellular and Integrative Physiology, Indiana University School of Medicine, Indianapolis, IN 46202, USA; 8 School of Computer Science and Technology, Harbin Institute of Technology, Harbin, Heilongjiang, 150001, China; 9 Information and Computer Engineering College, Northeast Forestry University, Harbin, Heilongjiang, 150001, China; 10 Division of Human Cancer Genetics, Ohio State University, Columbus, OH, 43210, USA; 11 Department of Molecular Virology, Immunology, and Medical Genetics, Ohio State University, Columbus, OH, 43210, USA; 12 Comprehensive Cancer Center, Ohio State University, Columbus, OH, 43210, USA; 13 Medical Sciences, Indiana University School of Medicine, Bloomington, IN, 47405, USA

## Abstract

**Background:**

Estrogens regulate diverse physiological processes in various tissues through genomic and non-genomic mechanisms that result in activation or repression of gene expression. Transcription regulation upon estrogen stimulation is a critical biological process underlying the onset and progress of the majority of breast cancer. Dynamic gene expression changes have been shown to characterize the breast cancer cell response to estrogens, the every molecular mechanism of which is still not well understood.

**Results:**

We developed a modulated empirical Bayes model, and constructed a novel topological and temporal transcription factor (TF) regulatory network in MCF7 breast cancer cell line upon stimulation by 17β-estradiol stimulation. In the network, significant TF genomic hubs were identified including ER-alpha and AP-1; significant non-genomic hubs include ZFP161, TFDP1, NRF1, TFAP2A, EGR1, E2F1, and PITX2. Although the early and late networks were distinct (<5% overlap of ERα target genes between the 4 and 24 h time points), all nine hubs were significantly represented in both networks. In MCF7 cells with acquired resistance to tamoxifen, the ERα regulatory network was unresponsive to 17β-estradiol stimulation. The significant loss of hormone responsiveness was associated with marked epigenomic changes, including hyper- or hypo-methylation of promoter CpG islands and repressive histone methylations.

**Conclusions:**

We identified a number of estrogen regulated target genes and established estrogen-regulated network that distinguishes the genomic and non-genomic actions of estrogen receptor. Many gene targets of this network were not active anymore in anti-estrogen resistant cell lines, possibly because their DNA methylation and histone acetylation patterns have changed.

## Background

Estrogens regulate diverse physiological processes in reproductive tissues and in mammary, cardiovascular, bone, liver, and brain tissues [1]. The most potent and dominant estrogen in human is 17β-estradiol (E2). The biological effects of estrogens are mediated primarily through estrogen receptors α and β (ER-α and -β), ligand-inducible transcription factors of the nuclear receptor superfamily. Estrogens control multiple functions in hormone-responsive breast cancer cells [2], and ERα, in particular, plays a major role in the etiology of the disease, serving as a major prognostic marker and therapeutic target in breast cancer management [2].

Binding of hormone to receptor facilitates both genomic and non-genomic ERα activities to either activate or repress gene expression. Target gene regulation by ERα is accomplished primarily by four distinct mechanisms (additional file 1) [3-5]: (i) ligand-dependent genomic action (i.e., direct binding genomic action or "DBGA"), in which ERα binds directly to estrogen response elements (ERE) in DNA. Candidate DBGA gene targets include PR and Bcl-2; (ii) ligand-dependent, ERE-independent genomic action (i.e., indirect binding genomic action or "I-DBGA"). In I-DBGA, ERα regulates genes via protein-protein interactions with other transcription factors (such as c-Fos/c-Jun (AP-1), Sp1, and nuclear factor-κB (NFκB)) [4]. Target I-DBGA genes include MMP-1 and IGFNP4; (iii) Ligand-independent ERα signaling, in which gene activation occurs through second messengers downstream of peptide growth factor signaling (e.g., EGFR, IGFR, GPCR pathways). Ligand-independent mechanism can be either DBGA or I-DBGA. These pathways alter intracellular kinase and phosphatase activity, induce alterations in ERα phosphorylation, and modify receptor action on genomic and non-genomic targets; (iv) rapid, non-genomic effects through membrane-associated receptors activating signal transduction pathways such as MAPK and Akt pathways (i.e. non-genomic action, NGA). Note that the term, non-genomic effect, is based on the fact that estrodial signaling pathway doesn't involve ERα itself (additional file 1) and as a consequence there is no direct ERα mediated transcription. Furthermore, target genes can receive input from multiple estrogen actions, e.g., cyclin D1 is a target of multiple transcription factors (TF): SP1, AP1, STAT5, and NFκB [3]. These four complex regulatory mechanisms, which describe the distribution of ERα and co-regulators in the nucleus and membrane signal transduction proteins, are called *topological mechanisms *and instrumental in sustaining breast cancer growth and progression.

Dynamic gene expression changes characterize the breast cancer cell response to estrogens, and the kinetics of ERα target genes are strongly influenced by the hormone treatment times. Early work by Inoue *et al. *[6] revealed distinct gene clusters that correspond to either early or late E2-responsive genes. Frasor and co-workers [7] defined "early" responsive targets in MCF7 cells as genes up- or down-regulated by 8 h after E2 treatment; genes induced by 24 h post E2 treatment were classified as "late" responders and can be blocked by the protein translation inhibitor cycloheximide. It was further demonstrated that cyclin D1 expression was mediated by the interaction of ERα-Sp1 (early response) and by MAPK-activated EIk-2 and SRF [3] (later response). As ERα binding sites are more significantly associated with E2 up-regulated rather than down-regulated genes [8], Carroll et al. hypothesized that physiologic squelching is a primary cause of early down-regulation and late down-regulation is an ERα-mediated event. Collectively, these studies and many others [9] strongly support a *temporal mechanism *of ERα regulation.

A number of gene regulatory network models have been developed to integrate ChIP-chip and gene expression data, including genetic regulatory module algorithm (GRAM) [10], statistical analysis of network dynamics (SANDY) [11], Bayesian error analysis model (BEAM) [12], and two-stage constrained space factor analyses [13-15]. Although a unified model framework was used to establish regulatory networks, those computational approaches were not capable of distinguishing genomic and non-genomic mechanisms, presumably due to failure to account for key differences in the type of data corresponding to genomic and non-genomic mechanisms. ERα genomic targets consist of protein binding signals (ChIP-chip peaks), which is not the case for non-genomic targets, and thus models and regulation selection for genomic and non-genomic ERα regulatory mechanisms are different. In addition, although the above computational approaches join models for ChIP-chip and gene expression data, TF motif scans are not typically performed, making it difficult to infer ERα DBGA or I-DBGA targets from these approaches.

In this study, we developed a new modulated empirical Bayes approach to assemble the ERα regulatory network. Our approach, for the first time, differentiates topological features of ERα regulation mechanisms: DBGA, I-DBGA, and NGA. By examining the estrogen-responsive gene network in breast cancer cell models, we established that the ERα regulatory network changes over time. This modulated empirical Bayes model controls false positives arising from ChIP-chip binding data, TF binding site (TFBS) motif scans, and differential gene expression profiles. Two applications of this regulatory network were studied. In the first application, the agonist/antagonist activities of two active metabolites of tamoxifen, 4-OH-tamoxifen and endoxifen, were investigated. The second application investigated the impact of epigenetics (DNA methylation and histone modifications) on ERα regulatory network in our previously established breast cancer cell model of acquired tamoxifen resistance [16].

## Results

### Data analyses overview

The ERα regulatory network model was developed based on differential gene expression data for MCF7 (untreated, 4 and 24 hour post E2 treatment) [16,17] and ERα ChIP-chip data [8]. The antagonistic/agonistic effects of OHT and endoxifen on this network were assessed using MCF7 gene expression microarray data at 24 hour post E2, OHT, endoxifen, E2+OHT, and E2+endoxifen treatments [17]. In MCF7 cells with acquired resistance to tamoxifen, the response of the ERα regulatory network was evaluated using gene expression microarray data [16], and the epigenetic mechanisms for non-responsive ERα network in MCF7-T cells were investigated by H3K4me2 and H3K27me3 ChIP-seq data and MCIp-seq.

### ERα regulation mechanisms and ERα targets

Based on ERα ChIP-chip data and microarray mRNA expression profiles after E2 stimulation of MCF7 breast cancer cells, we categorized ERα regulatory mechanisms into three groups (additional file 2): genomic action with ERα direct ERE binding (DBGA), genomic action with ERα indirect/ERE-independent (e.g., AP-1) binding (I-DBGA), and non-genomic/ligand-independent action (NGA). In DBGA, the activation of ERα can be either by E2 (ligand-dependent) or growth factor-mediated phosphorylation (ligand independent) (additional file 1 and additional file 2). Our current data is not able to distinguish between these two types of mechanisms.

**Figure 1:**
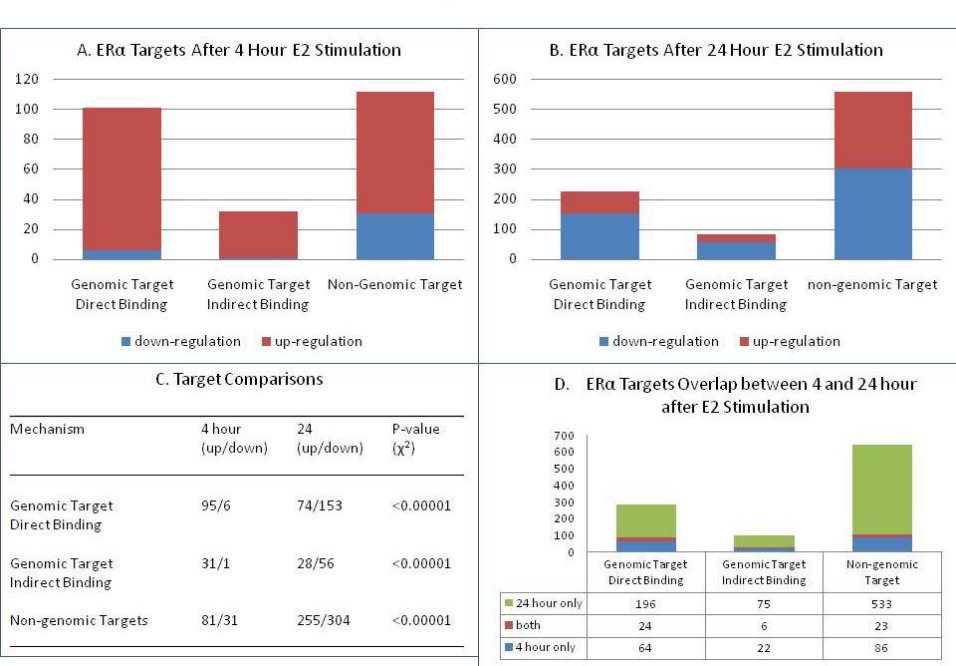
****Statistics of ERα targets after E2 stimulation**. **(A) ERα targets after 4 hour E2 stimulation in MCF7 cells; (B) ERα targets after 24 hour E2 stimulation in MCF7 cells; (C) Comparisons of up/down-regulated targets within each of three ERα regulation mechanisms; and (D) ERα targets overlap between 4 and 24 hour after E2 stimulation.

Different ERα mechanisms and their targets in MCF7 cell are displayed in Figure 1. For the three ERα mechanisms described above, more up-regulated targets were observed than down-regulated targets after 4 hour E2 stimulation (Figure 1A). Both DBGA and NGA mechanisms have more targets than I-DBGA has. After 24 hour E2 stimulation, a greater (p < 0.00001 vs. 4 hour) number of down-regulated targets was observed for all three mechanisms (Figure 1B &1C). These results are not totally consistent with the results in [8], as we use the 20% fold-change as an additional filtering criterion. Many significantly down-regulated genes have small fold change, especially after 4 hour E2 treatment.

It is interesting to note that the number of DBGA and I-DBGA targets at 24 hour was approximately doubled compared to 4 hour, while an approximate 5-fold increase in the number of NGA targets was observed at 24 hours (Figure 1A &1B). Furthermore, there was strikingly little overlap among the ERα targets between the two time points (8.5%, 5.8%, 3.8% for DBGA, I-DBGA, and NGA) respectively.

Gene ontology enrichment analysis was performed for the genomic and non-genomic targets at 4 and 24 hour after E2 stimulation, and the top 5 functional categories are listed in Table 1 (p-value range for sub-functional categories is reported for each category). Although both genomic and non-genomic mechanisms share only a small number of targets, their functions are highly consistent. At both 4 and 24 hours, genomic targets are mainly responsible for gene expression, cell morphology, cellular growth/development/movement, and cell cycle/death. On the other hand, at both time points, non-genomic targets are attributed to RNA post-translational modification, DNA replication/re-combination/repair, amino acid metabolism, cellular assembly and organizations. Therefore, genomic and non-genomic mechanisms have dramatically different impacts on the molecular and cellular functions in breast cancer cells.

**Table 1:** Gene Ontology Analysis of Estrogen Targets

ERα Target Mechanism	4 hour after E2 Stimulation			24 hour after E2 Stimulation		
	**Functional Category**	**P-value Range**	**N**	**Functional Category**	**P-value Range**	**N**

Genomic	Gene Expression	2E-6 - 9E-3	26	Cellular Growth	4E-7 - 1E-2	96
	Cell Morphology	4E-6 - 1E-2	15	Cell Cycle	2E-6 - 1E-2	37
	Cellular Growth	3E-5 - 1E-2	37	Cell Death	4E-5 - 1E-2	70
	Cellular Development	5E-5 - 1E-2	22	Cellular Movement	5E-5 - 1E-2	46
	Cell Cycle	1E-4 - 1E-2	21	Cellular Development	6E-5 - 1E-2	48

Non-genomic	RNA Post-Transcription	5E-6 - 4E-2	5	DNA Replication, Recombination, and Repair	1E-9 - 3E-2	62
	Modification					
	Cellular Development	8E-4 - 5E-2	2	Cell Cycle	1E-9 - 3E-2	70
	DNA Replication, Re-combination, and Repair	1E-3 - 4E-2	6	RNA Post-Transcription Modification	6E-6 - 2E-2	16
	Cellular Growth	1E-3 - 4E-2	8	Post-Transcription	5E-4 - 3E-2	15
	Amino Acid Metabolism	5E-3 - 5E-2	2	Modification Cellular Assembly and Organization	6E-4 - 3E-2	37

### ERα regulatory networks and their hubs

**Figure 2:**
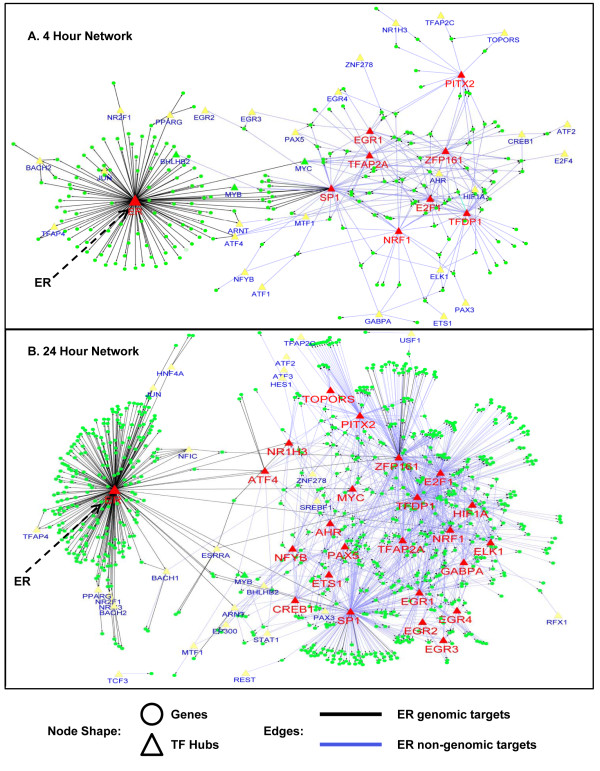
****ERα regulatory network after E2 stimulation**. **(A) ERα regulatory network after 4 hours E2 stimulation in MCF7 cells; and (B) ERα regulatory network after 24 hours E2 stimulation in MCF7 cells.

**Figure 3:**
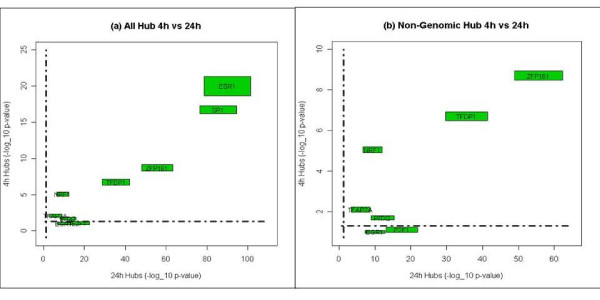
****Regularory hubs in ERα regulatory network**. **(A) The correlation of the significance of hubs between 4 hour and 24 networks; and (B) The correlation of the significance of non-genomic hubs between 4 hour and 24 networks. Both axis are the -log(p-value), and the width and length of the squares represent the relative scales of hubs.

After 4 hours of E2 stimulation, the ERα regulatory network is composed of an ERα hub and multiple interconnected hubs (Figure 2A). Both ERα (DBGA) and Sp1 (I-DBGA) hubs are consistent with genomic mechanisms, while the other hubs follow non-genomic mechanisms. The target sizes of genomic and non-genomics hubs are approximately equal; however, after 24 hour of E2 stimulation, there is a pronounced increase in the number of non-genomic hubs and targets compared to genomic hubs and targets (Figure 2B). These results demonstrate that while both genomic and non-genomic hubs are equally important, a greater number of late response E2 targets are activated through non-genomic mechanisms compared to genomic hubs. In addition, a striking feature of this dynamic ERα regulatory network is that a consistent set of transcription factors appear to control the hubs, despite the lack of overlap for hub targets between the two time points (discussed above; Figure 1D). These factors include (ZFP161, TFDP1, NRF1, TFAP2A, EGR1, E2F1, PITX2). Further comparison of the significant hubs between the 4 and 24 hour networks shows that both statistical significance (p-value) and hub size are consistent between two time points for both genomic and non-genomic hubs (Figure 3).

### Antagonistic/Agonistic effects of tamoxifen metabolites: 4-OH tamoxifen and endoxifen

Different SERMs have been shown to have different antagonistic/agonistic on E2 up- and down-regulated genes [18]. The effect of the tamoxifen metabolites OHT and endoxifen, both well-known SERMS [17], on ERα target networks has not been compared, particularly with regard to ERα genomic/non-genomic targets. Among the ERα targets identified after 24 hour of E2 stimulation, 17% and 14% were responsive to OHT and endoxifen respectively, with 74% of the targets overlapping (additional file 3). The agonist, antagonist, and partial agonist/antagonist activity of OHT and endoxifen on the ERα targets at 24 hour post E2 stimulation were nearly identical for the two SERMS (41%, 7%, 52% and 40%, 7%, 53% for OHT and endoxifen, respectively; additional file 4).

**Figure 4:**
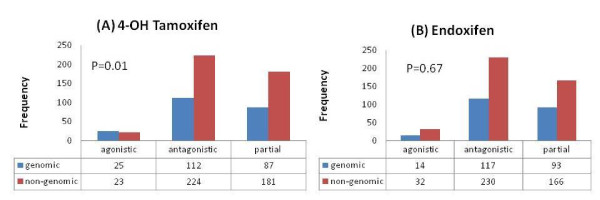
**Effect of selective ERα modulators**. (A) The agonistic effect of 4-OH tamoxifen is greater on genomic mechanism than on antagonistic or partial effects (p = 0.01). (B) No evidence for agonistic, antagonistic, or partial effects of endoxifen on genomic or non-genomics mechanisms.

We further classified the effects of OHT and endoxifen on ERα genomic/non-genomic and up/down regulation. There was a tendency for a greater agonistic effect on ERα genomic targets than non-genomic targets after E2 or OHT treatment (p = 0.01; Figure 4A). However, this difference in agonistic activity on genomic/non-genomic targets was not seen (p = 0.67, Figure 4B) after E2 or endoxifen treatment.

### Epigenetic modifications impact the ERα regulatory network in tamoxifen resistant MCF7 cells

**Figure 5:**
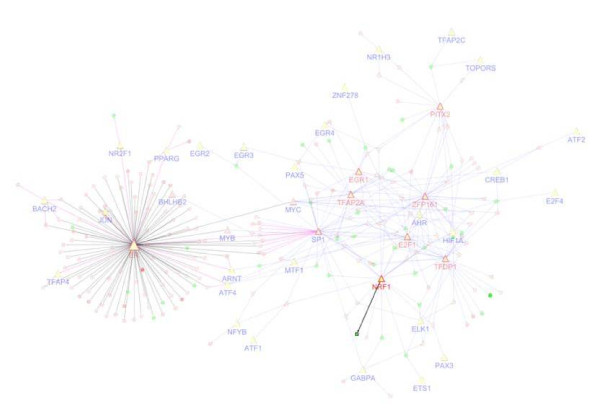
**ERα regulatory network in drug-resistant cells**. ERα regulatory network in MCF7 cell after 4 hour E2 stimulation becomes non-responsive to E2 in the MCF7-T cell (only one target gene remains responsive).

**Figure 6:**
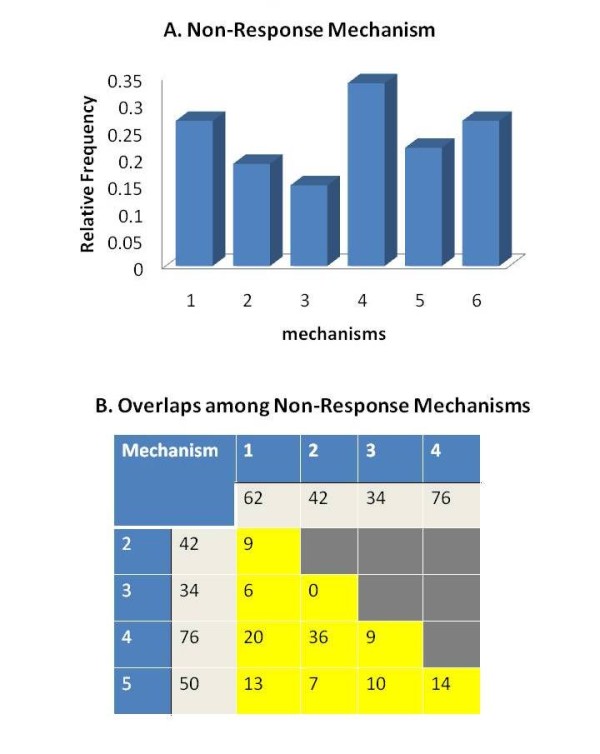
**Epigenetic mechanisms in drug-resistant cells**. Epigenetic mechanisms in ERα regulatory network in MCF7-T cell: 1 high basal gene expression in MCF7-T cells; 2 hypermethylation from MCF7 cells to MCF7-T cells; 3 hypomethylation from MCF7 cells to MCF7-H cells; 4 high basal methylation level in the MCF-T cells; 5 high H3K27/H3K4 ratio; and 6 unknown mechanisms. (A) The distribution of non-responsive mechanisms in ERα regulatory network in MCF7-T cell. (B) The overlap among 5 non-responsive mechanisms.

Breast cancer cell models for acquired resistance to tamoxifen display progressive loss of estrogen-dependent signaling for cell growth and proliferation and a disrupted ERα regulatory network [16]. Among the ERα targets observed after 4 hour E2 stimulation of MCF7, only one target remained hormone responsive in the tamoxifen-resistant MCF7-T subline (*NRF1; *Figure 5). In order to understand the role of epigenetics in this non-responsive ERα network, we investigated five possible mechanisms (additional file 5): (A) high basal gene expression in the MCF7-T cell; (B) hypermethylation (MCF7-T vs MCF7) (C) hypomethylation (MCF7-T vs MCF7); (D) high methylation level in MCF7-T; and (C) high H3K27/H3K4 ratio. As shown in Figure 6, these mechanisms account for approximately 27%, 19%, 15%, 34%, and 22% of the non-responsive targets (Figure 6A); however, these five mechanisms are not able to account for approx. 28% of targets. Substantial (36%) overlap was seen between hypermethylation (mechanism 2) and high basal methylation in MCF7-T cell (mechanism 4) (Figure 6B).

### Validation studies

*Pol II-Binding*. We compared PolII binding signals in MCF7 before and after 4 hour E2 stimulation. Nearly all ERα genomic targets displayed the same direction in fold-change between PolII binding and gene expression signals (98%; additional file 6A). Among the non-genomic targets, this concordance rate dropped slightly (86%). On the other hand, the concordance rate among non-targets was 55%.

*H3K4 Dimethylation *is a well established histone marker for transcription activation acetylation marker. We selected the median of H3K4 dimethylation ChIP-seq signal as the threshold. Almost all ERα genomic targets displayed H3K4 dimethylation higher than the median (94%, additional file 6B). Among the non-genomic targets, this concordance rate dropped slightly (84%). On the other hand, the concordance rate among non-targets was 49%.

*Overlap of 4 hour and 24 hour Estrogen Targets in the MCF7 Cell *We used a different data set by Cicatiello *et al. *[19], in which MCF7 cells were treated with E2, and sampled at baseline, 4 hr and 24 hr. This experiment was performed on a different gene expression platform, Illunima. We applied a similar empirical Bayes model and the same fold change threshold. We obtained a similar percentage of up/down regulated genes after 4h/24h estrogen treatment. In addition, the overlap of 4 and 24 hour gene targets was, 7%, similar to what we found out with our data.

*RT-qPCR, ChIP-PCR, and COBRA*. We further investigated four types of epigenetics mechanisms.

**Figure 7:**
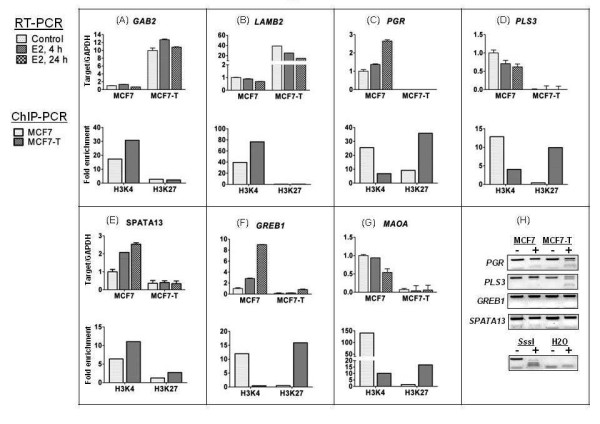
RT-PCR, ChIP-PCR and COBRA Validations

• Mechanism 1: GAB2 and LAMB2 were non-responsive in our network due to significantly increased basal expression in MCF7-T *vs*. MCF7 (based on microarray data). Although RT-qPCR analysis confirmed that GAB2 and LAMB2 expression was significantly higher in MCF7-T *vs*. MCF7 (Figure 7A,B), both genes were slightly responsive to E2 in MCF7-T. Our interpretation is that Affymetrix technology can be saturated for highly expressed genes, becoming insensitive to subtle expression changes. Nonetheless, the non-responsive mechanism needs further experimental investigation.

• Mechanism 5: PGR, PLS3, SPATA13, GREB1, and MAOA were non-responsive because of a high ratio of H3K27me3:H3K4me2 in MCF7-T *vs*. MCF7. Using ChIP-PCR, this mechanism was validated in four of five target genes (Figure 7C,D,F,G; exception was SPATA13, Figure 7E).

• Mechanisms 2 and 4: the DNA methylation status four ERα targets (PGR, PLS3, CREB1, SPATA13) was examined. Using COBRA assays, increased DNA methylation was observed in PGR and PLS3 in MCF7-T compared to MCF7 (Figure 7H; mechanism 4), and increased methylation in the MCF7-T and the MCF7 (mechanism 2). Furthermore, in the non-responsive ERα network, both PGR and PLS3 displayed both repressive epigenetic modifcations, the altered histone methylation ratio (mechanism 5) and altered DNA methylation (mechanism 2 and 4).

## Discussion

### Advantage of the modulated empirical bayes method in assembling a TF regulatory network model

Our proposed ERα regulatory network model framework differs from existing methods in its ability to distinguish between genomic and non-genomic actions, and the assumption for functional TFs. The pioneer TF regulatory network for *Saccharomyces cerevisiae*, developed by Luscombe *et al. *[11] and Lee *et al. *[20], emphasized that TFs themselves should be highly expressed and display differences in expression level. However, these assumptions tend to be overly stringent and not suitable for our data. Our gene expression microarray data suggested that the majority of the TFs (more than 70%) are expressed at low levels in MCF7 cells, and E2 stimulation results primarily in changes in TF phosphorylation state and not robust changes in TF expression in breast cancer cell lines, including MCF7 [7,16,21]. All of the TFs in our genomic and non-genomic hubs didn't change their expression significantly (additional file 7 and additional file 8). Stringent statistical models have recently been developed to establish TF regulatory networks [12,13,15]. Such regression-based approaches were not significant when used to analyze our data (not even for ERα itself), mainly due to the fact that TFs, including ERα, have both up- and down-regulated targets. If targets that change in opposite directions are not treated differently, the regression model will cancel-out any effect of a TF on gene expression. Therefore, regression model-based approaches to identify TF regulatory networks can be sensitive to a mis-specified model.

Our proposed empirical Bayes method modulates FDR calculations from differential gene expression data, ChIP-chip binding peaks, and TF motif scans. The inferred ERα regulatory network model has the following features and advantages:

• Distinct genomic and non-genomic mechanisms.

• Less stringent requirements on TF gene expression levels.

• Modulated data analysis leading to robust conclusions with respect to model misspecifications.

• Modulated model assembly results in an extendable TF network, which is particularly useful when additional data becomes available for new molecular mechanisms.

### ERα regulatory network and corresponding hubs

When constructing genomic targets of the ERα regulatory network, TFs are scanned within a narrow region, 45bp, of ERα ChIP-chip binding sites. This calculation scheme enables the identification of either DBGA or indirect I-DBGA. In many previous studies [8,22-24], relatively large neighborhoods surrounding the ERα binding site (around 500~1000bp) were scanned for consensus sequences of TFBSs. While this is an effective strategy for identifying co-regulatory TFs, it is not an effective approach for inferences regarding DBGA or I-DBGA. For example, Lin *et al. *[23] demonstrated that EREs and ERE half-sites were enriched for other transcription factors motifs, supporting the notion that TFs, in addition to ERα, can bind to ERE. In our analysis, we identified only Sp-1 as an I-DBGA. Although AP1 has been reported to be an I-DBGA, in our data it did not pass the false positive threshold (FDR = 0.23), due to its relatively short TFBS (6 bp). Binding motifs for forkhead TFs have also been reported to be enriched within ERα binding regions in MCF7 cells by ChIP-chip [8]. However, in our study, there was not sufficient evidence to support FoxA1 as an I-DBGA (FDR = 0.34), a result supported by recent studies using ChIP-seq and ChIP-DSL [25-27]. Recently, RAR and ERα binding were shown to be highly coincident throughout the genome, competing for binding to the same or similar response elements [28]. Our ERα regulatory network model, however, is not able to identify RAR targets, as the ChIP-chip experiments were only performed for ERα binding sites and not RAR.

In our analysis, non-genomic targets of the ERα regulatory network were constructed using genes whose promoters, introns, or downstream sequences were devoid of ERα ChIP-chip binding sites. Significant TF scan scores of these gene promoters infer ERα non-genomic action (NGA). It is worth noting that these NGA differ from previously described ERα co-regulator factors. NGA does not require ERα binding, in contrast to ERα co-regulatory factors which must display ERα binding peaks in the ChIP-chip analysis. Significant NGA transcription factors include ZFP161, TFDP1, NRF1, TFAP2A, EGR1, E2F1, and PITX2 (p <0.01). Other significant NGA includes MYC, which has been previously reported [28], and although MYC was present in both 4 and 24 hour ERα regulatory networks, the level of significance was not high enough to be considered a hub (p = 0.14).

Among the nine hubs that are significantly enriched in both 4 hour and 24 hour ERα networks, two facilitate genomic activities (ERα and Sp1), while the other seven hubs (ZFP161, TFDP1, NRF1, TFAP2A, EGR1, E2F1, PITX2) mediate non-genomic actions. With the exception of (ZFP161, TFDP1, PITX2), the functions of (Sp1, NRF1, E2F1, TFAP2A, EGR-1) and their functional relevance to estrogen action in breast cancer cells have been extensively documented in [29-32].

While the ERα regulatory network concept has recently been reviewed [33,34], our study is the first to characterize genomic and non-genomic mechanisms and their different functions. The genomic mechanism is significantly involved in cell proliferation and control of cell phases, confirming a significant effect of estrogen on cell cycle regulation. Biological processes significantly affected by the non-genomic mechanism include RNA post-translation modification, cellular development, DNA replication, re-combination, and repair. Additional models describing network properties of estrogen signaling targets include the protein-protein interaction and the functional module networks [28]. The focus of the two networks is on the functional interpretation of the targets and not mechanism of regulation. Furthermore, the edges are interpreted as either protein interaction or functional similarity and are not directional, compared to the edges in our regulatory network, which have up or down-regulation direction.

### Antagonist/agonist effects of SERMs on ERα regulatory networks

We observed full and partial antagonist/agonist effect of OHT on MCF7 after 24 hour E2 stimulation, similar to a previous study [18]. We further show that genomic and non-genomic actions of the ERα regulatory network are differentially influenced by full or partial antagonist/agonist activities of OHT and endoxifen. The current study clearly demonstrates that the E2 responsive ERα regulatory network is disrupted by two SERMs (additional file 4), but whether new networks are stimulated by these or other SERMs require additional investigation.

### Epigenetic Modifications of ERα Regulatory Network in the MCF7-T Cell

**Figure 8:**
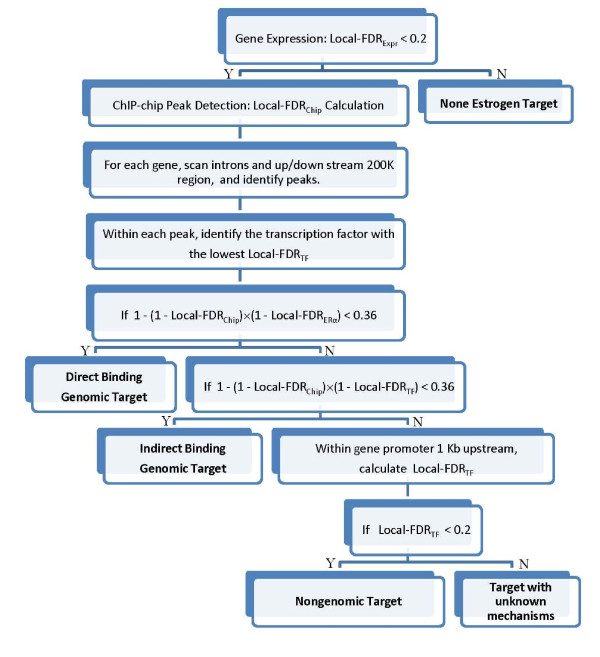
Flow-Chat of ERα Regulatory Network Construction

A second application of the regulatory network was to examine the impact of epigenetics (DNA methylation and histone modifications) on the ERα regulatory network in a breast cancer cell model for acquired tamoxifen resistance of [16]. Transcriptionally active genes are typically marked by higher levels of di-/tri-methylated H3K4 (H3K4me2/3) and low trimethylated H3 lysine 27 (H3K27me3) levels [35], and in hormone responsive MCF7 cells, E2-stimulated target genes have been shown to posses enriched regions of H3K4me1/2 [36]. In contrast, MCF7 with acquired tamoxifen resistance (MCF7-T), groups of previously E2-responsive genes are now associated with low H3K4me2 and high H3K27me3 and are either downregulated or no longer strongly hormone inducible (Figure 8). The H3K27me3 mark is stable and invariably associated with transcriptional repression [37,38] and we show that this repressive histone modification plays a key role in the unresponsive ERα regulatory network in MCF7 cells with acquired resistance to tamoxifen (Figure 8). Although tumorigenic gene silencing mediated by H3K27me3 has been shown to occur in the absence of DNA methylation [38,39], repressive histone marks frequently coordinate with the more permanent mark of DNA methylation in heterochromatin [39-41]. We previously demonstrated that alterations in DNA methylation play an important role in acquired tamoxifen resistance [16]. By integrating both repressive epigenetic marks into our model, we demonstrate that H3K27me3 and DNA methylation significantly contribute to the non-responsive ERα regulatory network model in tamoxifen resistant breast cancer. Furthermore, having recently demonstrated that many TFBSs are enriched in regions of altered DNA methylation [42], we suggest that the functions of activators or repressors could be altered by changes to the DNA methylation landscape and further impact ERα networks in breast cancer, an active area of investigation in our laboratory.

When we compare the percentages of different epigenetic mechanisms (Figure 7, 27%, 19%, 15%, 34%, 22%), to 20% each for a random gene set based on the selected thresholds, it seems that the non-responsive targets have similar distribution of various types of epigenetic mechanisms as that of a random gene set. Therefore, it is possible that there may not exist specific patterns of epigenetic mechanisms in MCF7 cells' acquired tamoxifen resistance.

## Conclusions

In breast cancer cells, we identified a number of estrogen regulated target genes and the estrogen-regulated network that characterizes the causal relationships between transcription factors and their targets. This network has two major mechanisms, the genomic action and the non-genomic action. In genomic action, after estrogen receptor is activated by estrogen, estrogen receptor regulated genes through directing binding to DNA. In non-genomic action, estrogen regulated its gene targets through non-direct binding through other factors. In the estrogen regulated network, we found that though many non-genomic targets change over time, they do share many common factors and the consistency is highly significant. Moreover, we found that many gene targets of this network were not active anymore in anti-estrogen resistant cell lines, possibly because their DNA methylation and histone acetylation patterns have changed. Taken together, our model has revealed novel and unexpected features of estrogen-regulated transcriptional networks in hormone responsive and anti-estrogen resistant human breast cancer.

## Methods

### Chromatin immunoprecipitation and ChIP-Seq library generation

Chromatin immunoprecipitation (ChIP) for PoI II (sc-899X, Santa Cruz, CA), H3K4me2 (Millipore, 07-030, Billerica, MA) and H3K27me3 (Diagenode, CS-069-100, Sparta, NJ) was performed as previously described [43]. ChIP libraries for sequencing were prepared following standard protocols from Illumina (San Diego, CA) as described in [44]. ChIP-seq libraries were sequenced using the Illumina Genome Analyzer II (GA II) as per manufacturer's instructions. Sequencing was performed up to 36 cycles for mapping to the human genome reference sequence. Image analysis and base calling were performed with the standard Illumina pipeline, and with automated matrix and phasing calculations on the PhiX control that was run in the eighth lane of each flow-cell. Samples were run on duplicates.

### Methyl-CpG immunoprecipitation (MCIp-seq)

MCIp-seq was performed and followed the manufacture's protocol (MethylMiner, Invitrogen, Carlsbad, CA). Briefly, genomic DNA was sheared by sonication into 200-600-bp fragments, and methylated DNA was immuno-precipitated by incubating 1 μg of sonicated genomic DNA for 1h at room temperature with 3.5 μg of recombinant MBD-biotin protein and Streptavidin beads. Methylated DNA was eluted with high-salt buffers (500 or 1,000 mmol/L NaCl), and then recovered by standard phenol chloroform procedure. The DNA fractions were subjected to library generation and followed by Illumina sequencing. Samples were run in duplicate.

### Quantitative ChIP-PCR

To determine binding levels of H3K4me2 and H3K27me3 on target genes, quantitative ChIP-PCR was used to measure the amount of this sequence in anti-H3K4me2 or H3K27me3-immunoprecipitated samples by PCR with SYBR Green-based detection (Applied Biosystems). Experimental quantitative ChIP-PCR values were normalized against values obtained by a standard curve (10-fold dilution, R^2^>0.99) constructed by input DNA with the same primer set. Specific primers for amplification are available upon request.

### Reverse transcription and quantitative PCR (RT-qPCR)

Total RNA (1 μg) was reverse transcribed with the Superscript III reverse transcriptase (Invitrogen, Carlsbad, CA). PCR was performed as described previously [45]. Specific primers for amplification are available upon request. The relative cellular expression of a coding gene was determined by comparing the threshold cycle (Ct) of the gene against the Ct of GAPDH.

### Identification of differentially expressed genes and FDR calculation

An empirical Bayes approach in the mixture-model framework was developed to assess differential gene expression data from Affymetrix platform. Because the differential expression inference is made at the gene level rather than at the probe level, our model is an extension of Kendziorski's work [46,47]. In this model, between-gene variation, between-probe variation and between replicate are included. Specifically, let *i *index genes (*i *= 1.2.,...,*I*), l index conditions/groups/time (*l *= 1,2; 1 is the reference), *j *index probe set (*j *= 1,2,..., *n*_*i*_) and k index replicate (*k *= 1,2,..., *m*_*i*_). Let *G*_*ijk *_be the expression level of the *k*th replicate on probe *j *for gene *i *under group *l*. We consider the following random-effects model:(1)

where *μ*_*il *_is the gene expression level for gene *i *under condition *l*,*b*_*ij *_represents the probe effect for the *j*th probe of gene *i *and *ε*_*ijkl *_is the error term (for genes with only one probe, the probe effect *b *is eliminated from model (1)). We consider that the genes come from three latent populations, each of which is characterized by the location of *μ*_*ij *_(X variable) and *μ*_*i2 *_(Y variable) on a two-dimensional plane. The first population, a bivariate normal distribution with the center located above the y = x line, represents up-regulated genes. The second population, a normal distribution along y = x line, represents unchanged genes. The third population, a bivariate normal distribution with the center below the y = x line, characterizes down-regulated genes. Denote by *Y*_i _a latent indicator such that *Y*_*i *_= 1,0,-1 implies that gene *i *belongs to the first, second and third populations, respectively. Thus, we consider the following model for *μ*_*il*_:(2)

where *I*(.) is a function that takes value 1 if the argument is logical/true and 0 if otherwise; *BN *and *N *denote the bivariate and univariate normal distributions, respectively. By integrating equations (1) and (2), one can use the Expectation-Maximization (EM) algorithm (S1.doc) to estimate the parameter vector **θ **= (**ρ**, **η**_**1**_, **Σ**_**1**_, **η**_**-1**_, **Σ**_**-1**_,*λ*,φ,**σ**,**δ**). The posterior probability  can be interpreted as the probability that gene *i *is not differentiated. Rigorously speaking,  cannot be directly interpreted as the probability that gene *i *is up/downregulated. However, a probability close to 1 indicates a good approximation. In our analysis, we claim that a gene is up-regulated if  and  or downregulated if  and . The local FDR can be easily estimated by  or [48]. In our analysis, we set *c *= 0.80. Models (1) and (2) are fitted to baseline and E2 stimulated (4 and 24 hours) expression data for MCF7 cells. In addition to FDR, we also set 20% fold-change in either up- or down-regulation in expression as the biologically significant effect size. Binding Scores for Peak Areas Identified by ChIP-chip and FDR Calculation is based on model-based analysis of tiling-arrays [49].

### Motif binding site scan and FDR calculation

*Genomic Binding Sites: *Each significant ChIP-chip peak binding site sequence of length 45 bp (25 bp of tiling array probes plus 10 bp up/downstream of each probe) is scanned by all of the TF motifs in TRANSFAC databases. The range of binding scores for a transcription factor with motif *M *are divided into a number of small bins (*k *= 200). The number of scores fall into each bin is then calculated. If the number of any bin is lower than a pre-specified limit (*t *= *m*_*b *_20), the bin is collapsed with neighboring bins until the number is beyond the limit. The number of scores that fall in each bin is denoted *b *by *m*_*b*_. Then, we randomly generate *R *= 10,000 sequences based on human genome background using a 6^th ^order Markov model. This model assumes that a sequence element probability depends on 6 previous bases, immediately preceding the current base [50]. The binding scores for these random sequences are calculated, and the number of scores that falls into each bin is denoted by *n*_*b*_. Finally, the local FDR, in terms of binding event for scores in bin *b*, is calculated as(3)

where *I *is the total number of genes. In doing so, we force the bins below the midpoint of the score range to have *FDR*_*b,m *_= 1 because it is highly unlikely that these low score bins represent true binding events. Finally, we fit a cubic smoothing-spline to *FDR*_*b,m *_to get *FDR*_*s,m*_, the local FDR at score *s *(degree = 4, # of knots = # of unique *FDR*_*b,m *_values). Then for each gene, we have the FDR estimate respect to the event that TF *g *binds to gene *i's *promoter. This non-parametric approach to estimate FDR was first described by Efron *et al. *[51] in differential gene expression data analysis.

*Non-genomic Binding Sites: *We applied the same method as above to the motif binding scores collected from each gene promoter upstream 1Kb.

### Modulated empirical bayes model: DBGA, I-DBGA, and NGA mechanism determination based on ChIP-chip peak, TF motif scan and differential gene expression data

Based on FDRs calculated from empirical Bayes models in differential gene expression, ChIP-chip binding peaks, and TF motif scan scores, DBGA, I-DBGA, and NGA targets were calculated using the flow-chart displayed in Figure 8. Graphical interpretations of different mechanisms and their associated data types are displayed in Figures S1 and S2. In brief, both genomic and non-genomic targets must have significantly differentially expressed genes, while only genomic targets have significant ChIP-chip binding peaks. Finally, a DBGA has a significant ERα motif in the ChIP-chip binding sites, an I-DBGA has one or more significant TF motifs (other than ERα) in the ChIP-chip binding sites, and a NGA has one or more significant TF motifs in its target gene promoter.

### TF Hub significance calculation

To quantify the significance of well-connected TF hubs, we consider the following null hypothesis: TFs that are involved in the regulation of differential genes are randomly picked from a pool of known TFs. Specifically, we suppose there are *M *differential genes. For each gene *i*, there are *bi *binding sites by ChIP-chip and motif search that pass the threshold, which involve *n*_*i *_(*n*_*i *_≤ *b*_*i*_) unique TFs. Therefore, there are a total of  involved TFs. If there are *n *known TFs, then under the null hypothesis the number of connected nodes for each TF is the same as the number of times each TF appear from *M *random draws with each draw of size *n*_*i*_. Note that each draw of *n*_*i *_is without replacement because they represent distinct transcription factors. The distribution of the number of connected nodes (_*T*_) for any TF is(4)

where Ω(*t*) is the set of all subsets of {1,2,...,*M*} with *t *elements. Hence, p-values associated with hub TFs can be obtained by calculating Pr(*T *≥ *t*_*obs*_), where *t*_*obs *_is the observed number of genes regulated by the TF of interest. This calculation is programmed in R.

### Signal identification for ChIP-seq (PolII, H3K4me2, H3K27me3) and MCIp-seq

In order to evaluate transcriptional activity, activating and repressive histone methylation marks, and DNA methylation of ERα target genes, ChIP-seq data for RNA Pol II, H3K4me2, and H3K27me3 and MIRA-seq data DNA methylation were analyzed. Total sequences were normalized among replicates. For the ChIP-seq data, the signal intensity was measured as the number of ChIP-seq tags within the promoter region, defined as 1,000-bp upstream of TSS (transcription start site). In the MCIp-seq data, seq tags within upstream 1000bp and downstream 1000bp of the TSS were selected for promoter DNA-methylation.

### Identifications of agonist, antagonist, and partial agonist/antagonist selective estrogen receptor modulator (SERM) targets

Let (*FC*_*E*2_, *FC*_*SERM*_, *FC*_*E*2+*SERM*_) be the fold-change of gene expression after treatment of MCF7 cells with E2, SERM (OHT or endoxifen), or E2+SERM). We defined fold-change as gene expression in the treatment group over the control group for up-regulation; otherwise, it is defined as the minus inverse ratio. In particular, if a gene is absent in both groups, the fold-change is defined as 1. A SERM has an agonistic effect on a gene if |*FC*_*SERM*_| > [1 + 70% × (|*FC*_*E*2_| - 1)], an antagonistic effect if |*FC*_*SERM*_| < [1 + 35% × (|*FC*_*E*2_| - 1)] and |*FC*_*E*2+*SERM*_| < [1 + 50% × (|*FC*_*E*2_| - 1)]; otherwise, it has a partial agonistic/antagonistic effect. These agonist and antagonist activities have been defined previously [18].

### Epigenetic mechanisms of non-responsive ERα network in 4-hydroxy tamoxifen (OHT) resistant MCF7 cells

For ERα targets in the ERα regulatory network 4 hours after E2 stimulation, five different epigenetic mechanisms were investigated (additional file 5).

• The first mechanism (additional file 5A) is the high-basal gene expression in the 4-OHT-resistant MCF7 cells, in which the threshold of high-basal gene expression is defined as its 80^th ^percentile.

• The second mechanism (additional file 5B) is defined as the hyper-methylation: *i.e*., higher methylation level of OHT-resistant MCF7 than the parental (hormone-responsive) MCF7. The threshold of this fold-change is defined as its 80^th ^percentile.

• The third mechanism (additional file 5C) is defined as the hypo-methylation: *i.e.*, lower methylation level of OHT-resistant MCF7 *vs*. MCF7. The threshold of this fold-change is defined as its 80^th ^percentile.

• The fourth mechanism (additional file 5D) is defined as the high methylation in the OHT-resistant MCF7. The threshold of methylation level is defined as its 80^th ^percentile.

• The fifth mechanism (additional file 5E) is defined as the high H3K27/K3K4 ratio, a gene repressive mark, in the OHT-resistant MCF7. The threshold of this ratio level is defined as its 80^th ^percentile.

All other non-responsive ERα targets were categorized as "unknown".

## Abbreviations

ERα: estrogen receptor α; TF: transcription factor; E2: 17-estradiol; MCF7: luminal-like breast cancer cells; OHT: 4-OH tamoxifen; ERE: estrogen response elements; TFBS: TF binding site; EM: Expectation-Maximization;

## Authors' contributions

CS designed part of the computational study, implemented the empirical Beyes model and drafted part of the manuscript. YH designed the validation study, conducted the experiments to validate the computational model, and drafted part of the manuscript. YL designed part of the computational study, implemented part of the analysis of ChIP-chip data and motif search, and drafted part of the manuscript. GW, YZ, ZW and MT implemented part of the analysis of ChIP-chip data and motif search and drafted part of the manuscript. YW, DF and TS provided critical guidance on the computational and experimental elements of the study and made critical revision of the manuscript. PY carried out part of validation experiments and revised the manuscript, KN and TH provided biological guidance on the interpretation of the computational model and design of the validation experiments, and drafted part of the manuscript. LL conceived the overall design of the study, drafted most part of the manuscript, and provided both statistical and biological input for the development and validation of the computational model. All authors read and approved the final manuscript.

## Supplementary Material

Additional file 1 is a jpeg file, indicating the situations of ligand-dependent genomic target, ligand-independent genomic target and non-genomic target Click here for file

Additional file 2 is a jpeg file, indicating the relationships between data and ERα mechanisms Click here for file

Additional file 3 is a jpeg file, indicating the effect of 4OH-tamoxifen and endoxifen on the network Click here for file

Additional file 4 is a jpeg file, indicating agonistic, antagonist, and partial agonistic/antagonistic effects of 4-OH-tamoxifen and endoxifen Click here for file

Additional file 5 **is a jpeg file, indicating non-responsive mechanisms in ERα regulatory network in MCF7-T cell**. (A) high basal gene expression in MCF7-T cells; (B) hypermethylation from MCF7 cells to MCF7-T cells; (C) hypomethylation from MCF7 cells to MCF7-H cells; (D) high basal methylation level in the MCF-T cells; (E) high H3K27/H3K4 ratio. Click here for file

Additional file 6 **is a jpeg file, indicating the concordance between differential PolII bindings and differential gene expression among genomic-targets, non-genomic targets, and none targets; and the concordance between H3K4 dimethylation among genomic-targets, non-genomic targets, and none targets**. (A) The concordance of differential gene expression and PolII binding are before and after E2 stimulation of MCF7 cells. (B) The concordance of differential gene expression and H3K4 dimethylation. Click here for file

Additional file 7 Supplementary Table 1 Click here for file

Additional file 8 Supplementary Table 2 Click here for file
