# Supplementary figures and images for: A modulated empirical Bayes model for identifying topological and temporal estrogen receptor α regulatory networks in breast cancer
Source: BMC Syst Biol. 2011 May 9;5:67. doi: 10.1186/1752-0509-5-67 (PMC3117732; doi:10.1186/1752-0509-5-67)

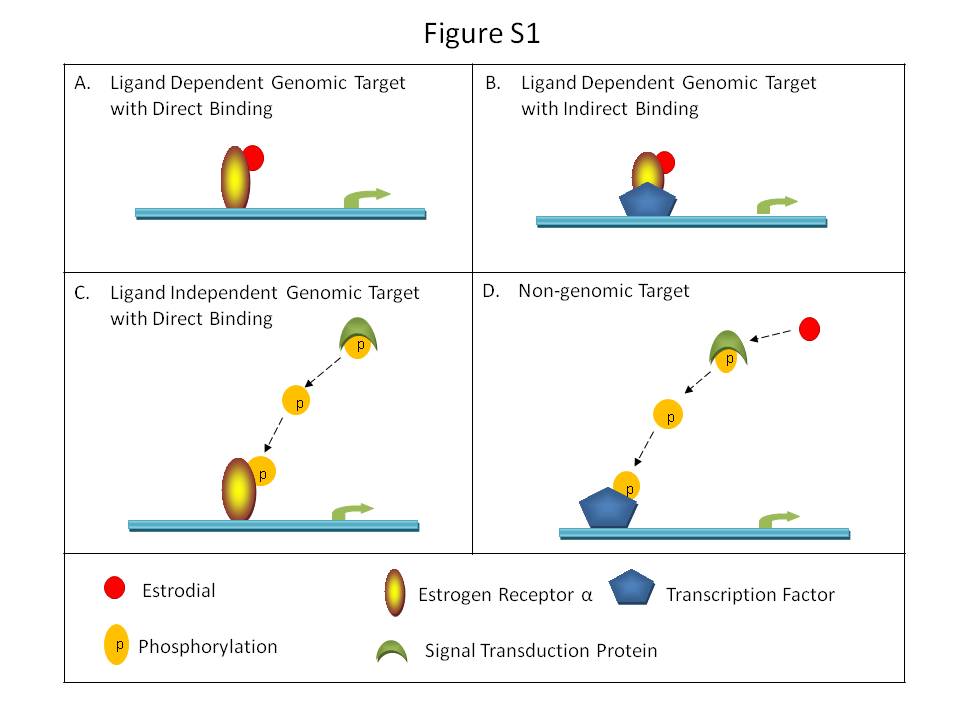

Supplement: Additional file 1 — is a jpeg file, indicating the situations of ligand-dependent genomic target, ligand-independent genomic target and non-genomic target [file 1752-0509-5-67-S1.JPEG]

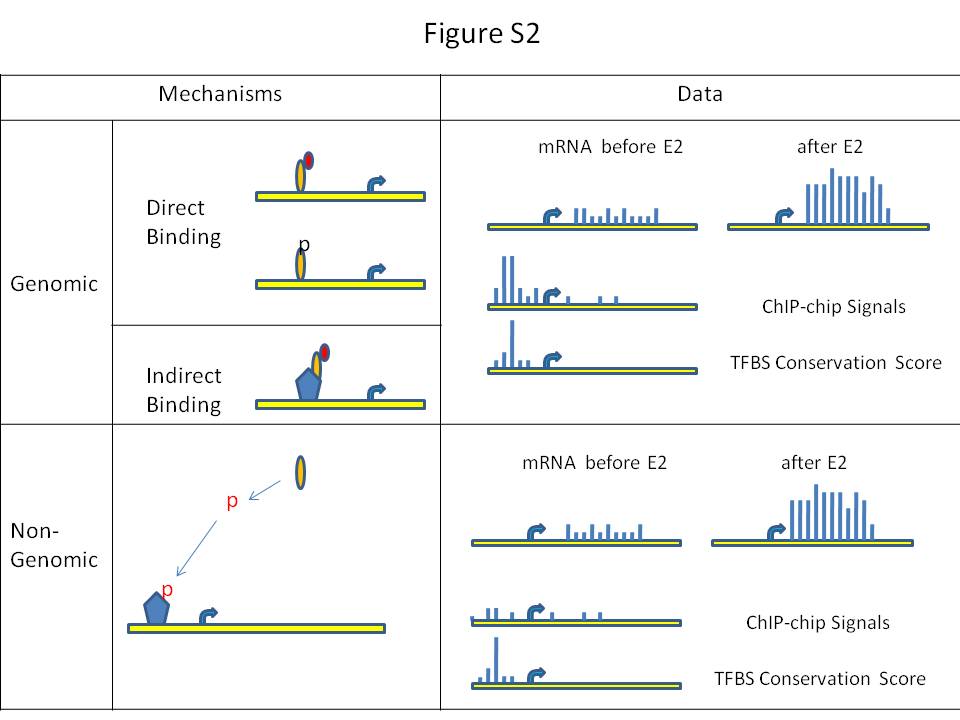

Supplement: Additional file 2 — is a jpeg file, indicating the relationships between data and ERα mechanisms [file 1752-0509-5-67-S2.JPEG]

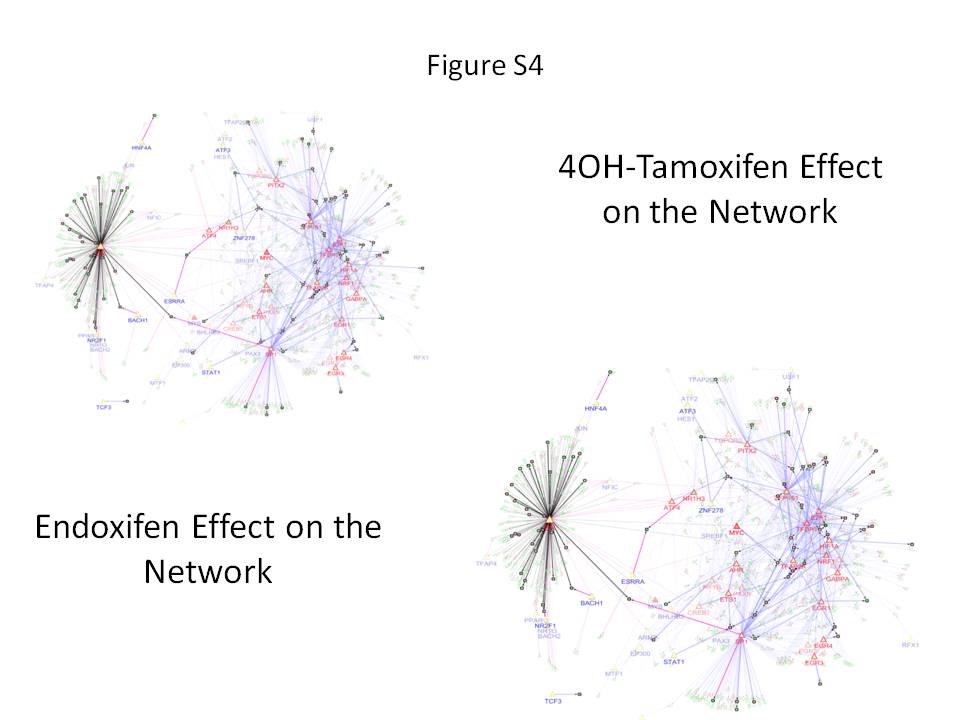

Supplement: Additional file 3 — is a jpeg file, indicating the effect of 4OH-tamoxifen and endoxifen on the network [file 1752-0509-5-67-S3.JPEG]

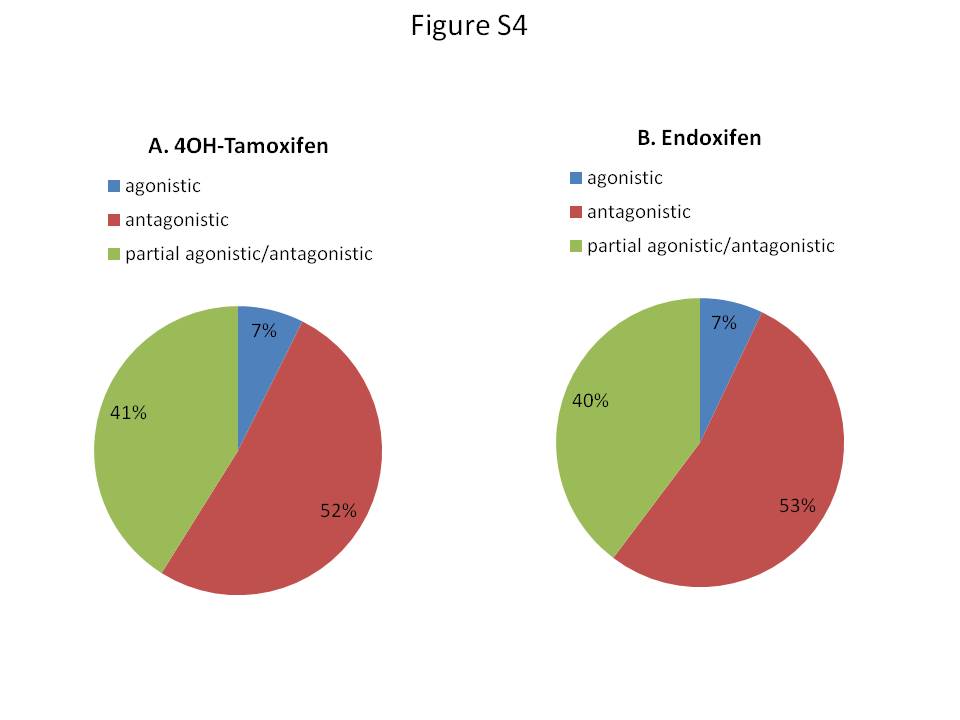

Supplement: Additional file 4 — is a jpeg file, indicating agonistic, antagonist, and partial agonistic/antagonistic effects of 4-OH-tamoxifen and endoxifen [file 1752-0509-5-67-S4.JPEG]

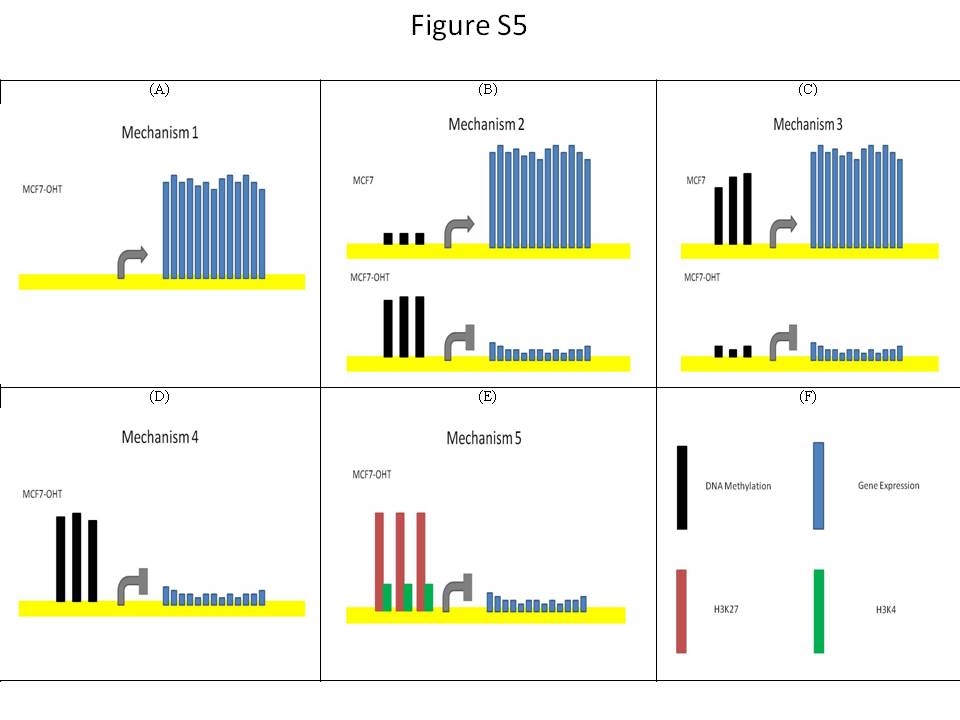

Supplement: Additional file 5 — is a jpeg file, indicating non-responsive mechanisms in ERα regulatory network in MCF7-T cell. (A) high basal gene expression in MCF7-T cells; (B) hypermethylation from MCF7 cells to MCF7-T cells; (C) hypomethylation from MCF7 cells to MCF7-H cells; (D) high basal methylation level in the MCF-T cells; (E) high H3K27/H3K4 ratio. [file 1752-0509-5-67-S5.JPEG]

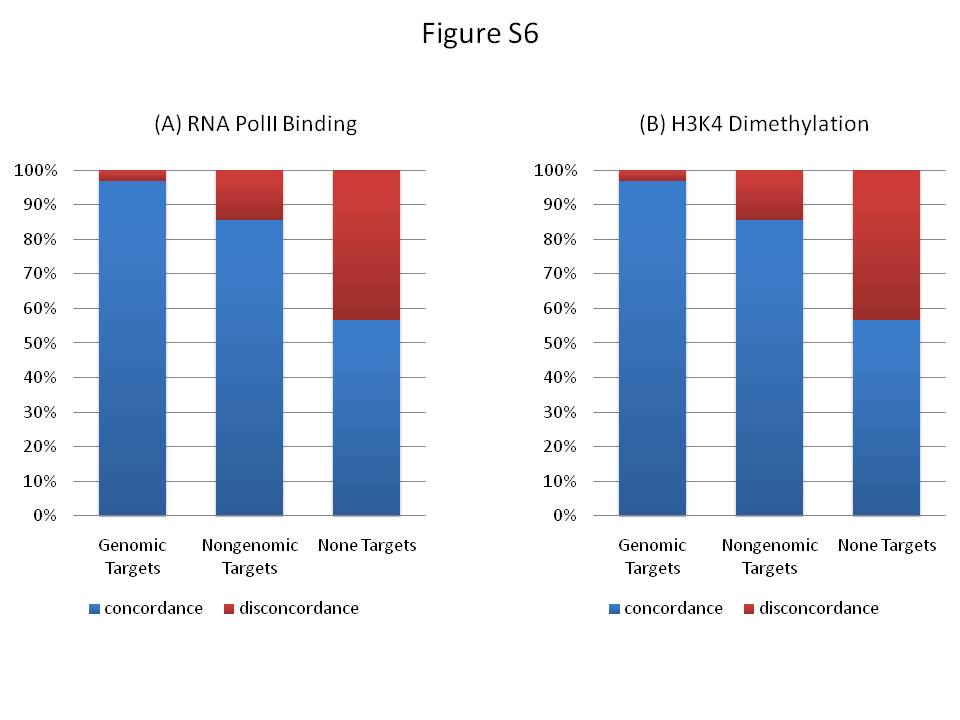

Supplement: Additional file 6 — is a jpeg file, indicating the concordance between differential PolII bindings and differential gene expression among genomic-targets, non-genomic targets, and none targets; and the concordance between H3K4 dimethylation among genomic-targets, non-genomic targets, and none targets. (A) The concordance of differential gene expression and PolII binding are before and after E2 stimulation of MCF7 cells. (B) The concordance of differential gene expression and H3K4 dimethylation. [file 1752-0509-5-67-S6.JPEG]
